# Perceptions about penis size among supposedly healthy 40 to 60-year-old Brazilian men: a cross-sectional pilot study: Percepções sobre o tamanho do pênis em homens brasileiros supostamente saudáveis de 40 a 60 anos: um estudo piloto transversal

**DOI:** 10.1590/1516-3180.2013.7710008

**Published:** 2014-09-26

**Authors:** Margareth de Mello Ferreira dos Reis, Sidney Glina, Carmita Helena Najjar Abdo

**Affiliations:** I PhD. Psychologist at Instituto H. Ellis and Researcher at the Department of Psychiatry, Faculdade de Medicina da Universidade de São Paulo (FMUSP), São Paulo; and Coordinator of the Postgraduate Course “New Paradigms in Sexual Health” at Faculdade de Medicina do ABC, Santo André, Brazil.; II MD, PhD. Head of the Department of Urology, Hospital Ipiranga, São Paulo, Brazil.; III MD, PhD. Program of Studies on Sexuality (ProSex), Department and Institute of Psychiatry, Faculdade de Medicina da Universidade de São Paulo, São Paulo, Brazil.

**Keywords:** Erectile dysfunction, Sexuality, Body dysmorphic disorders, Penis, Prevalence

## Abstract

**CONTEXT AND OBJECTIVE:**

Many men seek medical treatments complaining that their penises are too small (short) when in fact they are not (they are not cases of micropenis). The objective of the present study was to evaluate men’s satisfaction with their own bodies and sex life and the prevalence of erectile dysfunction, among men who were not seeking medical or psychological advice.

**DESIGN AND SETTING:**

Cross-sectional study in a private, philanthropic hospital in São Paulo, Brazil.

**METHODS:**

In this study, 300 male blood donors aged between 40 and 60 years old answered a questionnaire, in privacy, about their sex life and their satisfaction with their own bodies. They were also screened for erectile dysfunction by means of the International Index of Erectile Function questionnaire.

**RESULTS:**

Seven men (2.3%) reported that they were dissatisfied with their penis size (they thought that it was small), and among these, one was found to have mild erectile dysfunction. However, none of them had sought medical attention. Among these seven, only two had normal body mass index; the other five were overweight (three) or obese (two).

**CONCLUSION:**

The prevalence of dissatisfaction with penis size was low. Among the seven dissatisfied men, only one had erectile dysfunction, of mild type, and all of them felt potent.

## RESUMO

**CONTEXTO E OBJETIVO:**

Muitos homens buscam tratamento médico com a queixa de que seus pênis são muito pequenos (curtos), quando na verdade não são (não são casos de micropênis). O objetivo do presente estudo foi avaliar a satisfação com o próprio corpo e vida sexual e a prevalência de disfunção erétil entre homens que não estavam buscando aconselhamento médico ou psicológico.

**TIPO DE ESTUDO E LOCAL:**

Estudo transversal, em um hospital privado filantrópico em São Paulo, Brasil.

**MÉTODOS:**

Neste estudo, 300 homens doadores de sangue, com idade entre 40 e 60 anos, responderam um questionário, em privacidade, sobre sua vida sexual e satisfação com o próprio corpo. Eles também foram rastreados para disfunção erétil por meio do questionário International Index of Erectile Function.

**RESULTADOS:**

Sete homens (2,3%) disseram estar insatisfeitos com o tamanho de seus pênis (achavam que era pequeno), e entre estes, um tinha disfunção erétil leve. Entretanto, nenhum deles procurou atendimento médico. Entre esses sete, somente dois estavam com índice de massa corporal normal, três estavam com sobrepeso e dois eram obesos.

**CONCLUSÃO:**

A prevalência de insatisfação com o tamanho do pênis foi pequena. Entre os sete homens insatisfeitos, apenas um tinha disfunção erétil, leve, e todos se sentiam potentes.

## INTRODUCTION

The penis is considered to be a symbol of masculinity in many cultures, and the phallus often represents potency, fertility, strength and male power. Phalluses are often represented in ancient and modern pictures and sculptures in many regions of the world. Penis size is given much importance, especially by men, and it is commonly cited as an attribute of hegemonic masculinity.^1^^,^^2^

In recent years, patients seeking treatments for what they call “small penis” have sought urologists more and more frequently.^2^ A Google search for “penile enlargement”, on July 2, 2011, retrieved more than 19 million websites, thus showing indirectly that there is great popular interest in gaining increased penis size. Micropenis is a medically described condition of a penis of less than 4 cm (flaccid) or 7 cm (stretched).^3^^,^^4^ This abnormality of penis size is also considered to be 2.5 standard deviations smaller than the mean penile length for a given population.^5^ “Candidates” for penile augmentation would be those with a length of less than 4 cm (flaccid) or 7.5 cm (erect/stretched).^4^ Normal penis size has been measured in several studies and is known to be different according to the population observed. In a review by Ghanem et al., average penis length was reported as being 12.3 cm stretched and 12.7 cm erect.^6^

However, most of the men (or parents bringing children) seeking help for “small penises” do not really present abnormal penis sizes.^6^^,^^7^ They are just esthetically dissatisfied,^6^ and many surgery clinics (urology and plastic) are probably profiting from this dissatisfaction. While this is an issue still under investigation, this complaint has already being named in the literature as “penis dysmorphophobia”,^3^^,^^6^^,^^7^ a condition in which men seek medical treatments believing that their penises are too short.^3^^,^^7^ Some studies have shown that, on measurement, their penises are in fact found to be normal.^3^^,^^6^^,^^7^^,^^8^^,^^9^ Once informed that they have no abnormality, approximately 70% of these men give up treatment.^7^^,^^8^ The fact is that their penises are not impairing sexual activity (intercourse), because they are normal sized.

What would be the beliefs of men who have not sought medical advice for penis enlargement? We recently communicated the results from a cross-sectional study on the prevalence of erectile dysfunction in men who considered themselves healthy (they were not recruited in hospitals or clinics, and they were healthy enough to be blood donors) and who were not seeking diagnoses or self-information on sexual behavior or function. That study^10^ revealed an opportunity to investigate whether those men were satisfied with their penis size. The present study is thus a specific analysis on the previous database.

## OBJECTIVE

The aims here were to investigate: 1) the prevalence of dissatisfaction with penis size, the whole body and sex life, among those men who were considered healthy (blood donors) and who were not seeking treatment; and 2) whether men dissatisfied with their penis size would also suffer from erectile dysfunction, as defined through the International Index of Erectile Function. The hypothesis was that there would be cases of erectile dysfunction among men who were dissatisfied with their penis size.

## METHODS

### Study design

In this cross-sectional study, male blood donors were contacted in the waiting room of a private, philanthropic hospital in São Paulo, Brazil, between January 2006 and July 2007. The hospital’s Ethics Committee approved the study and all participants signed informed consent forms. To be eligible, the blood donors had to be 40 to 60 years old, with at least four years of schooling (total length of school attendance). Being heterosexual and in a stable partnership for at least six months, irrespective of marital status (in order to ensure a minimum period of sexual interaction with their partner), were also inclusion criteria. Those unable to understand or answer the questionnaires and men using medication that affects sexual functioning, such as diuretics, antidepressants and hypertension therapy, were excluded. After excluding some participants based on these criteria, the sample was made up of 300 subjects.

Data on weight, height and blood pressure were compiled from the subjects’ blood donation medical records. The men completed self-applied questionnaires in a single sitting: an identification form (for sociodemographic information), a questionnaire on erectile function and psychiatric screening. The presence of erectile dysfunction was evaluated using the International Index of Erectile Function, which had previously been transculturally adapted to Brazilian Portuguese.^11^ They also answered a specific question about their self-perception of erectile dysfunction: “do you feel sexually potent”? They were asked if they had sought for treatment for any problem they might have. The results from this analysis have already been published.^10^ The men also gave answers to questions about their own perception of aspects of their personal lives and bodies, such as sexual life, length of relationship with their partner, satisfaction, attraction towards their partner, sexually potency, erection and the satisfaction with their own body and penis size.

The subjects answered the questionnaires voluntarily while waiting to donate blood (and after being considered able to donate blood by health professionals), and without the help or the presence of the researchers. They had privacy to respond, and anonymity was guaranteed.

The frequency of dissatisfaction among the men regarding their whole body, penis size, sex life and erectile dysfunction was registered, as were their beliefs about sex. The profiles of the dissatisfied men were analyzed. The analysis was descriptive, presenting the frequencies of each personal characteristic or positive response to questions. No statistical test was applied because of the small size of the subsample.

## RESULTS

As already reported elsewhere,^10^ among the 300 men included, 236 (78.6%) were aged 49 years or less. The majority (213; 71%) had 4 to 11 years of schooling. The majority (274; 91.3%) were satisfied with their relationships, classifying them as good or excellent, and most (97.3%) felt sexually attracted towards their partners, and answered that their sexual desire was excellent or good (279; 93%). The majority of the men said that they felt comfortable when talking about sex. Twenty of them said they had a curved penis. Although the majority (253) said that they were satisfied with their bodies, most of them were overweight (body mass index, BMI ≥ 25 kg/m^2^; 200).

Seven men said that they were not satisfied with their penis size. Among these seven, only two had normal BMI, the other five were overweight (three) or obese (two). Three men were not satisfied with their bodies and they all felt “fat”: two were in fact obese and one was overweight. Only one of these dissatisfied men (their profiles are described below) had erectile dysfunction as defined through the International Index of Erectile Function, with a score of 21, indicating mild erectile dysfunction. He was obese and said the reason for being unhappy with his penis size was that he did not know what a normal size would be. All of them responded that they felt potent.

### Men’s profiles

Seven subjects (# 15, 26, 102, 171, 209, 227 and 233) were dissatisfied with their penis size. Their profiles (Table 1) are described individually below.

**Table 1.**
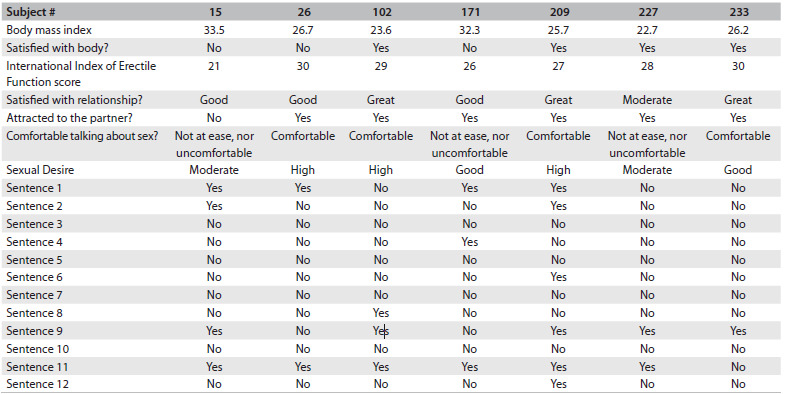
Data and responses to questions among seven men who were dissatisfied with their penis size

Sentence 1 - We are liberated people and we feel comfortable about sex; Sentence 2 - A real man does not dwell on things as delicate as feelings and communication; Sentence 3 - All physical contact is sexual or should lead to the sexual act; Sentence 4 - A man is always interested in and constantly willing to have sex; Sentence 5 - A real man performs well in the sexual act; Sentence 6 - Sex is centered on an erect penis and on what you do with it; Sentence 7 - Sex is the same as intercourse; Sentence 8 - A man should be able to make the earth shake under the feet of his partner; Sentence 9 - A good sexual act requires orgasm; Sentence 10 - Men should not listen to women during sex; Sentence 11 - Good sex is spontaneous, without planning and without talking about it; Sentence 12 - Real men do not have sexual problems.

Subject # 15: This obese man (BMI 33.56 kg/m^2^) presented erectile dysfunction that was classified using the International Index of Erectile Function questionnaire as mild erectile dysfunction (he obtained a satisfactory erection in most of his sexual encounters). This was the only man who reported having penile curvature, but he had never asked for medical advice about this. He felt uncomfortable with his body, because he was overweight. He was satisfied with the relationship with his partner, but had not felt sexually attracted to her for the last two years. In his opinion, his sexual desire was now “moderate”, and it had been like that for three years.

Subject # 26: This overweight man (BMI 26.70 kg/m^2^) thought that he was fat, with a waist that was too large. He desired a bigger penis. This man felt potent (he did not have erectile dysfunction according to the International Index of Erectile Function). He was satisfied with his relationship, and felt attracted to his partner. He said that he was comfortable talking about sex.

Subject # 102: This man had a normal weight for his height (BMI 23.66 kg/m^2^). He was very satisfied with his partner, to whom he was attracted; he felt potent (he did not have erectile dysfunction according to the International Index of Erectile Function) and thought that his sexual desire was “high”. He was satisfied with his body, but he thought that his penis was “small”. He responded that he felt comfortable talking about sex.

Subject # 171: This obese man (BMI 32.36 kg/m^2^) was satisfied with his partner and felt attracted to her. He felt potent (he did not have erectile dysfunction according to the International Index of Erectile Function) and felt sexual desire. He was not satisfied with his body and said that he was “fat”. He considered that his penis was “small”. He did not feel ashamed talking about sex, but neither did he feel comfortable about it.

Subject # 209: This slightly overweight man (BMI 25.77 kg/m^2^) was highly satisfied with his partner, to whom he felt attracted. He felt potent (he did not have erectile dysfunction according to the International Index of Erectile Function) and classified his sexual desire as “high”. He said that he felt “ok” talking about sex.

Subject # 227: This man with normal weight (BMI 22.75 kg/m^2^) was moderately satisfied with his relationship. The reason that he gave for this was that his family was feeling insecure about a possible transfer to another country, because their children had already moved out. He felt attracted to his partner, felt potent (he did not have erectile dysfunction according to the International Index of Erectile Function), but felt that his sexual desire had been “moderate” for the last two years. Nevertheless, he had not sought medical advice. He was satisfied with his body but not with his penis, which he thought was “small”. He did not feel ashamed talking about sex, nor was he comfortable.

Subject # 233: This man was slightly overweight (BMI 26.23 kg/m^2^). He was satisfied with his body, but not with his “small” penis. He was highly satisfied with his relationship and he felt attracted to his partner. He felt potent (he did not have erectile dysfunction according to the International Index of Erectile Function) and his sexual desire was good. He felt “ok” talking about sex.

## DISCUSSION

An Italian study assessed 67 men who visited an andrology clinic complaining of a short penis. The majority were concerned only about the length of the flaccid penis. They were asked to “guess” what a normal penis size would be and, for them, a penis length of 10 cm to 17 cm (12 cm on average) was ideal; 85% overestimated the normal penis size. However, 15% had no idea of what a normal penis size should be. None of the subjects had anatomical abnormalities or erectile dysfunction. The majority of them started to be concerned during childhood, when they felt their penises were shorter than those of their school colleagues, or during adolescence, when they began to watch erotic movies. A nomogram of the sample was constructed and none of the men was found to be below the average size. After being informed of this, 70% of the patients gave up the idea of having surgical treatment.^7^

Three years later, the Egyptian urologist Shamloul^8^ also asked his patients what the normal penis size should be, before measuring their size. They estimated that the normal size was 13 cm (range: 11 cm to 17 cm); 94% overestimated the normal penis size. None of them had erectile dysfunction or anatomical abnormalities such as a micropenis. The onset of worries about penis size began during childhood or adolescence for the majority. After an explanatory session about anatomy and sexual intercourse, 86% of the patients agreed that their penis size concerns had been eliminated. The remaining 14% received psychological counseling, after which 84% of these men gave up the idea of seeking enlargement surgery.

The data presented here were collected as part of a larger study of ours.^10^ The results presented here showed that 2.3% of the sample of blood donors said that they were dissatisfied with their penis size. These findings were obtained at a time when it was no longer possible to contact the subjects, who were interviewed at the time when they were in the blood center making their donations.

Therefore, our study did not measure penis size or make any physical evaluation: we only asked for men’s opinions about their own bodies. Thus, it was not possible to verify whether they had real reasons for concern or any detectable clinical/anatomical problems. Nor was it possible to psychologically evaluate whether these men were simply dissatisfied with an esthetic feature or whether they were really suffering from a “phobia”, i.e. a mental disorder characterized by an “imaginary defect” or an “obsession”.

While simple esthetic problems (such as big or small breasts or noses; or too much or too little hair) can be fixed successfully by means of esthetic surgery, penis enlargement is a complex operation with somewhat unpredictable results. The men described in this study, despite being dissatisfied with their penis sizes, had not sought medical or psychological help, nor had they informed themselves about what a normal penis size should be or obtained a solution for their problem. It would have been necessary to evaluate them individually to ascertain whether the problem was only mild esthetic discomfort (such as “I do not like my nose” or “I feel bad about being bald”), which is something that people can cope with over a long life, or whether it was something that led to distress.

It would be premature to say that the men in this study needed treatment, but on the other hand, their profiles suggest that this finding might have been associated with general dissatisfaction with the whole body, and not only the penis. These men would probably have benefited from referral for psychological evaluation.

Over the past year, several studies on men’s normal penis size were published, providing average length and circumference measurements.^2^^,^^4^^,^^12^^,^^13^^,^^14^ Some of them investigated correlations between penis size and height, BMI or other somatometric parameters, including index finger length.^12^^,^^13^^,^^14^ However, it is still too soon to establish an average penis size for each average height range or any other characteristic, since no significant associations were found. In fact, Lever et al.^15^ investigated this issue using the internet, with more than 50,000 participants, and found that 12% thought that their penises were small, while 22% thought that their penises were large and 55% said that they were satisfied with their penis size. Among the men who rated their penis size as “average”, 46% wanted it to be larger, and this rate increased to 91% among those who thought that their penises were small.

Another insight that can be obtained is that, as shown by other studies, because the idea of “normal penis size” varies according to the population, the cultural characteristics of the men should be taken into consideration. Brazilians might be more (or less) demanding about penis size than other populations, and only a larger study would be able to confirm whether this 2.3% prevalence of dissatisfaction would be representative of the national population. Phalloplasty would, in this context, be an individual solution for a cultural problem. Exposure to pornography should also be investigated, since it certainly gives many people nowadays a visual idea of penis size and function.^15^ Whether this idea would be realistic or not is an issue to be discussed further.

As stated by Lever et al.,^15^ “Addressing the problem of male dissatisfaction with penis size is particularly important in the modern technological age where alteration of the body through cosmetic surgery has become a widespread phenomenon”. Treating penises that are not really small can be considered to be esthetic therapy, rather than functional therapy.^3^ A recent review on the subject concluded: “Current data regarding the results and complication rate of interventional augmentation procedures are reported mainly in patients without an objective penile-shaft problem, and they are extremely disappointing. There is a need for scientific and methodological research on the outcomes and complication rate of all these procedures”.^9^ The review points out that, from the surgical point of view, the techniques available fail to show efficacy and the complication rate is high: infections, shortening (instead of increasing the length), curvature and retraction are some of the complications reported.

Because of the lack of standardization of clinical study reporting, descriptions of the complications may be lacking in many of the published articles. Ghanem et al.^6^ agreed that “penile augmentation surgery is still experimental and should be limited to research or university institutions with supervisory ethics committees, where well-informed, properly evaluated and properly counseled patients accept the potential risks of the procedure. Only limited data support the use of stretching devices for penile augmentation”.

As shown by the Italian study,^7^ reassurance about normality can avoid unnecessary treatments. We strongly agree with the idea that psychological evaluation and counseling can help patients before they even consider undergoing procedures that are always risky, such as surgery, or before they start buying useless penis enlargement devices sold through the internet. Rather, it is important to understand the factors that contribute towards penis dissatisfaction.^15^

The possibility that complaints of small penis size might be associated with erectile dysfunction was not confirmed in the present study. Only one of the dissatisfied men had erectile dysfunction as defined through the International Index of Erectile Function, and he presented a score of 21, indicating mild erectile dysfunction. He was obese and said that the reason for being unhappy with his penis size was that he did not know what a normal size should be (an issue that could be easily be resolved through a medical consultation).

All of the men interviewed responded that they felt potent, i.e. that their penis size was not interfering with erection. It is interesting to observe some paradoxical findings about their responses: firstly, although all of these seven men declared that they did not feel uncomfortable talking about sex, none had ever sought specialist advice about their dissatisfaction with their penis size. Two felt moderate sexual desire and moderate satisfaction in their relationships and one had no sexual attraction towards his partner at all. Nevertheless, none had sought medical or psychological counseling.

Reassurance work can be performed based on discussion of the common myths about sex that are spread around the population and which may contribute towards individuals’ dissatisfaction with their body and sex life. Zilbergeld, in his book “The new male sexuality”,^16^ commented on penis size saying that “size matters”. He stated that although penis size is a very common concern for men, they do not see each other’s erect penises except in erotic movies. What they see in these films are actors who have been hired precisely on the basis of uncommonly big penises, which are further enhanced through filming techniques such as lighting, camera tricks and other effects.

Thus, most men really do not have a realistic basis for comparison, and this was shown by both the Italian and the Egyptian study.^7^^,^^8^ The dissatisfaction among those subjects began during childhood and adolescence and, once they had been told that they were within the normal range, most of the men became reassured and gave up the idea of augmentation surgery.

What healthcare professionals should be aware of is that psychological counseling is helpful in restoring the quality of the sexual life of these dissatisfied men, and that a psychological clinical evaluation can also rule out other problems that may have been hidden, such as body dysmorphic disorder. These may be physically normal men with psychological complaints that may require evaluation, and these findings should be disseminated among the medical and psychotherapy communities, so that healthcare professionals can challenge patients’ beliefs about the association between penis size and masculinity.^15^

The use of blood donors as the subjects for the present study was considered to be an alternative to using urology clinic patients, healthcare service users or volunteers, who may be more prone to suffering from erectile dysfunction and other health problems than the general population (selection bias resulting from their interest in seeking treatment). These male blood donors were at least theoretically healthy and, most importantly, they were not seeking treatment for penis enlargement or sexual problems. Nonetheless, some of them (2.3%) were dissatisfied with their penis size.

Some studies have actually measured penis size among men in different populations. Data is already available in Brazil for comparison. A recent Brazilian study identified penis length among boys aged 0 to 18 years. The study was undertaken among 2010 subjects and found that the real length of the flaccid penis (fully stretched manually) was a consistent measurement, and 145 mm (with a standard deviation of 16 mm) was the average found for 18-year-old boys.^5^

Given the intimate nature of some of the questions involved in such studies, it is possible that subjects feel more comfortable providing answers on their own rather than directly to a researcher. This is the reason why our choice of a self-applied research instruments may have brought reliable results about penis size perception.

On the other hand, the present study was developed in the form of a cross-sectional study, which does not allow causal inferences between the outcomes studied and the characteristics of the subjects in the study (for example, body mass index and dissatisfaction with the body). One limitation of this study was the age of the men who were evaluated (40 to 60 years old); perhaps a younger population would have had a higher rate of dissatisfaction with their penis size. Nevertheless, this study provides some evidence that dissatisfaction with penis size may be an overlooked problem with a possibly unnoticed disorder.

## CONCLUSION

1. The prevalence of dissatisfaction with penis size among healthy middle-aged men was low. 2. Erectile dysfunction was not common among the men who were dissatisfied with their penis size. 3. The majority of the men who were dissatisfied with their penis size felt potent and sexually attracted to their partners, but they were overweight or obese, and this was a matter of concern to them.
